# DISCHARGE LOCATIONS AFTER HOSPITALIZATIONS INVOLVING OPIOID USE DISORDER AMONG MEDICARE ENROLLEES

**DOI:** 10.1093/geroni/igac059.1447

**Published:** 2022-12-20

**Authors:** Patience Moyo, Kimberly Goodyear, Eric Jutkowitz, Kali Thomas, Andrew Zullo

**Affiliations:** Brown University School of Public Health, Providence, Rhode Island, United States; Brown University School of Public Health, Providence, Rhode Island, United States; Brown University, Providence, Rhode Island, United States; Brown University, Providence, Rhode Island, United States; Brown University School of Public Health, Providence, Rhode Island, United States

## Abstract

Hospitalizations involving opioid use disorder (OUD) are increasing in the Medicare population. Rising OUD-related acute care use emphasizes the need to understand where post-acute care is provided and opportunities to facilitate OUD treatment in those settings. We conducted a retrospective cohort study using 2016-2018 Medicare Provider Analysis and Review (MedPAR) files linked to enrollment data and the Residential History File (RHF) for 100% of Medicare fee-for-service beneficiaries aged ≥18 years. We used diagnostic codes for opioid dependence or “abuse” to identify OUD from MedPAR data. The RHF identified hospital discharge locations. Our analysis included 459,763 OUD-related hospitalizations. Overall, individuals < 65 years (60.3%), female (53.7%), or Medicare-Medicaid dually enrolled (59.1%) were the majority. The mean (standard deviation) length of stay was 5.5 (6.6) days and 31.3% of OUD-related hospitalizations required intensive care use. Seventy percent of patients with OUD-related hospitalizations were discharged home, 15.8% to skilled nursing facilities (SNFs), 9.6% to non-SNF institutional facilities, 2.5% home with home health services, and 1.8% died in-hospital. Within 30 days of hospital discharge, the range for 30-day all-cause readmissions was 27.7% (home) to 47.5% (non-SNF institutional setting). The 30-day all-cause mortality ranged from 3.1% (home without health) to 6.4% (non-SNF institutional settings). Given that over one-quarter of OUD-related hospitalizations resulted in discharge to locations other than home and that many institutional post-acute care settings cannot obtain OUD medications, modifying Drug Enforcement Administration regulations and expanding access to OUD medications for aging adults in post-acute and long-term care settings must be a near-term policy focus.

